# Life history and biology of *Fascioloides magna* (Trematoda) and its native and exotic hosts

**DOI:** 10.1002/ece3.1414

**Published:** 2015-03-04

**Authors:** Miriama Malcicka

**Affiliations:** Department of Ecological Sciences, Animal Ecology, VU University AmsterdamDe Boelelaan 1085, Amsterdam, 1081HV, The Netherlands

**Keywords:** Definitive host, intermediate host, parasite, phylogeny

## Abstract

Host–parasite interactions are model systems in a wide range of ecological and evolutionary fields and may be utilized for testing numerous theories and hypotheses in terms of both applied and fundamental research. For instance, they are important in terms of studying coevolutionary arms races, species invasions, and in economic terms the health of livestock and humans. Here, I present a comprehensive description of the life history, biogeography, and biology of the giant liver fluke, *Fascioloides magna*, and both its intermediate and definitive hosts. *F. magna* is native to North America where it uses several species of freshwater snails (Lymnaeidae) as intermediate hosts and four main species of ungulates as definitive hosts. The fluke has also been introduced into parts of Europe where it is now established in two lymnaeid snail species and three ungulate species. This study gives a comprehensive description of different developmental stages of the fluke in its two host classes, as well as detailed notes on historical and present distributions of *F. magna* in North America and Europe as well as in its snail and deer hosts (with range maps provided). Aberrant and dead-end hosts are also discussed in detail, and descriptive phylogenies are provided for all of the organisms. I briefly discuss how *F. magna* represents a model example of multiple-level ecological fitting, a phenomenon not yet described in the empirical literature. Lastly, I explore possible future scenarios for fluke invasion in Europe, where it is currently expanding its range.

## Introduction

Host–parasite interactions have long proven to be model systems in testing numerous theories and hypotheses in both applied and fundamental ecological and evolutionary research. The giant liver fluke, *Fascioliodes magna* (Bassi, 1875), is an economically important trematode parasite of both domestic and wild ungulates that is native to North America (Pybus 2001). The fluke has a complex life cycle that includes intermediate snail hosts (Lymnaeidae) and definitive mammalian hosts (ungulates) (Erhardová-Kotrlá 1971; Špakulová et al. 2003a,b). Although this fluke species has been well studied in the context of pathological effects on different species of (mostly) definitive hosts (Foreyt and Todd 1976; Craig and Huey 1984; Stromberg et al. 1985; Conboy et al. 1988; Pybus 1990), much less is known about its biology and ecology in relation to the phylogeny or distribution of either its intermediate or definitive hosts. Moreover, it has been largely ignored in the invasion ecology literature, despite the fact that it has had to overcome a number of ecological and evolutionary hurdles to become established in parts of Central Europe, where it is currently established and expanding its range along Danube River (Kráľová-Hromadová et al. 2011). The number of suitable intermediate and definitive hosts for *F. magna* is limited, even in its native range (Pybus 2001). There it is known to use several species of snails during its life cycle and up to four wild ungulate species during the latter stages of its life cycle. In both instances, transmission from the intermediate to the definitive host is also dependent upon the presence of suitable wetland habitats where both occur together at the same time at least during some parts of the year (Pybus 2001). Immature parasites enter the body of the snail via the foot, whereas the next stage of its life cycle is physically attached to succulent vegetation and is ingested by grazing deer (Erhardová-Kotrlá 1971).

The first transport of fluke-infected wapiti to Europe in the 19th century did not enable the parasite to establish there, because of physical barriers (fences); however, the second introduction (and probably the third) to Europe in 20th century was successful in spreading the parasite's infection within Europe (Kráľová-Hromadová et al. 2011). Given the strong coevolutionary history between *F. magna* and its hosts, it is not surprising that North American intermediate and definitive hosts share a strong phylogenetic affinity with their European equivalents (Pitra et al. 2004; Correa et al. 2011). In Europe, it is known that at present, only two species of intermediate host have currently been described in nature, along with three species of definitive hosts under natural conditions (Špakulová et al. 2003a,b). The habitats frequented by its hosts in both continents are similar, as are some of the climatic conditions. In combination, these have enabled *F. magna* to establish and spread in Europe. Eradication programs are unlikely to succeed, given that it is now common in countries such as Austria, the Czech Republic, Slovakia, Hungary, Croatia, and Serbia (Majoros and Sztojkov 1994; Prosl 2001; Ursprung 2001; Vodňanský and Rajský 2001; Marinculić et al. 2002; Marinkovic and Nesic 2008; Rehbein et al. 2012).

Here, I present a comprehensive description of the life history, biology, and phylobiogeography of the giant liver fluke, *Fasciolides magna*, and both its intermediate and definitive hosts. This study gives a comprehensive description of different developmental stages of the fluke in its two host classes, as well as detailed notes on historical and present distributions of *F. magna* in North America and Europe as well as in its snail and deer hosts (with range maps provided). Particular attention is paid to comparing the ranges of the fluke relative to those of its definitive hosts in both continents in a phylobiogeographical framework. Aberrant and dead-end hosts are also discussed in detail, including the economic consequences of fluke infection in livestock. Moreover, descriptive phylogenies are provided for all of the organisms, in particular how physiologically equivalent abiotic and biotic conditions in North America and Europe facilitated successful invasion by the fluke into the latter. I briefly discuss how *F. magna* represents a model example of multiple-level ecological fitting, a phenomenon not yet described in the empirical literature. Lastly, I explore possible future scenarios for fluke infection in Europe, where it is currently expanding its range.

## A Model Parasite *Fascioloides magna*

The first description of this parasite was by Bassi (in Italy), 1875 as *Distomum magnum*. Between 1882 and 1892, the fluke was recorded from different areas of the United States and described separately by many authors. It has been known as *Distomum hepaticum* Curtice 1882; *Fasciola hepatica* Dinwiddie 1889 (nec. Linnaeus 1758); *Fasciola carnosa* Hassall 1891; *Fasciola americana* Hassall 1891; *Distomum texanicum* Francis 1891; *Cladocoelium giganteum* Stossich 1892. Later, Stiles (1894) pointed out that the American findings are identical with the species described previously by Bassi (1875). Stiles made a complete morphological description of the adult fluke and named it *Fasciola magna* (Bassi 1875) (Stiles 1894). Lastly, Ward (1917) established a new genus involving only one species, *Fascioloides magna* (Bassi 1875) (Ward 1917). Currently, it is commonly called the giant liver fluke, large American liver fluke, and deer fluke (Pybus 1990).

**Figure 1:**
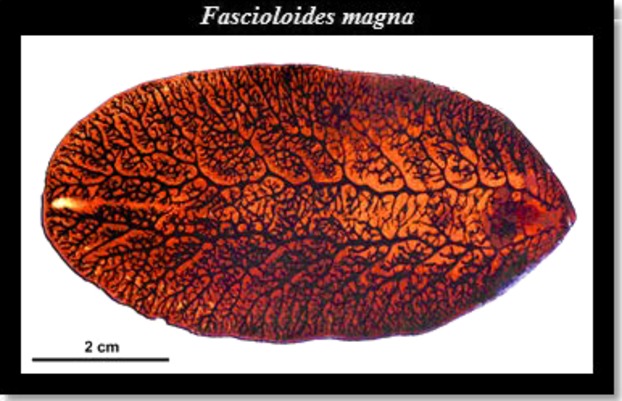
Adult stage of the giant liver fluke, *Fascioloides magna*. The photo was taken by Martin Kašný.

*Fascioloides magna* (Fig.1) is a multicellular parasite belonging to phylum Platyhelminthes, class Trematoda. In that phylum are also found eucestodes, monogeneans, and other minor groups, which are important in terms of in veterinary and human parasitology. Trematodes are characterized by a one-way gut, external tegument, protonephridia, and one or more external holdfast organs. Most of them are hermaphroditic, and complete male and female reproductive systems occur in each individual. Usually, they infect more than one host, including one or more intermediate hosts such as water snails or insects and vertebrate definitive hosts. The life cycle is heteroxenous, and mostly of them are endoparasites.

Giant liver flukes infect a large spectrum free-living animals (deer, moose, reindeer) and domestic ruminants (cattle, sheep, goat) (Pybus 2001). In definitive hosts, the parasites occur in the liver parenchyma and feed on blood. Unlike other liver flukes, *Fascioloides magna* is found directly in pseudofibrosis cysts in the liver. Effects of this parasite depend on the host type (Foreyt and Todd 1976). In definitive hosts (cervids), infection may proceed subclinical (e.g., nonlethally), but in dead-end hosts (goat, sheep), it may cause mortality. However, to complete the life cycle, the parasite must go through the intramolluscan stages (Erhardová-Kotrlá 1971), in a water snail of the genus *Galba* (Lymnaeidae).

**Figure 2:**
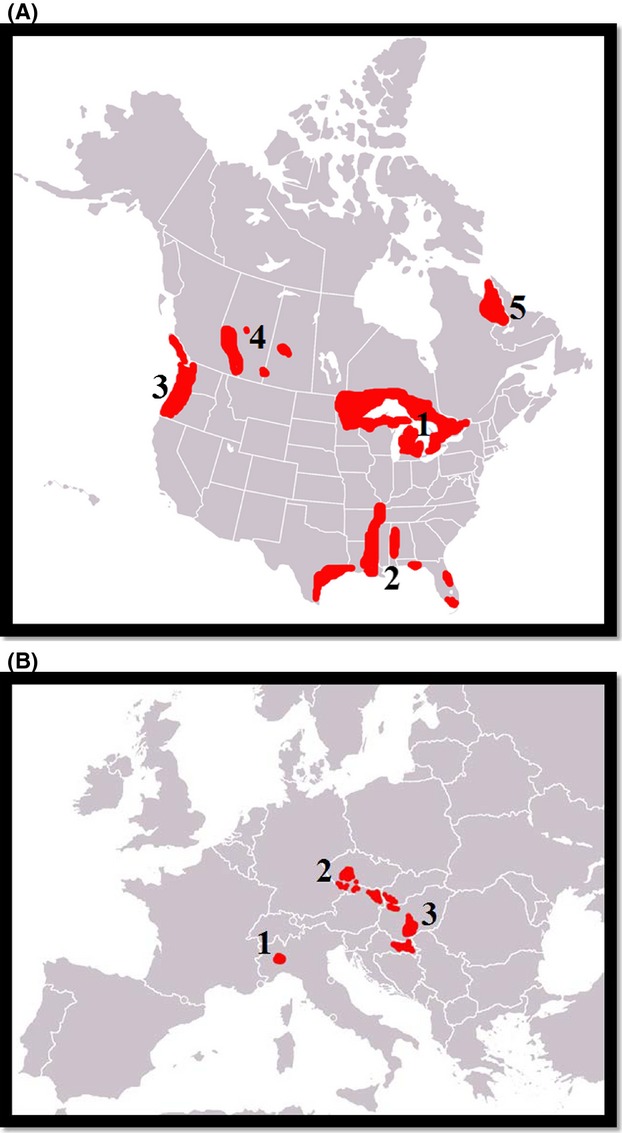
(A) Currently known native distribution of the giant liver fluke in its native range in North America based on Pybus (2001). The distribution is broken into five foci. (B) Currently known distribution of the giant liver fluke in its invasive range in Europe. The distribution is broken down into three defined foci (Špakulová et al. 2003a,b).

*Fascioloides magna* is native in North America, and it occurs throughout the United States and in southern parts of Canada, being most common in the northeastern U.S. and in states neighboring the Great Lakes (Lankester and Luttich 1988; Mulvey 1991). Currently, the fluke is enzootic in five major areas: (1) the Great Lakes region; (2) the Gulf coast, lower Mississippi, and southern Atlantic seaboard; (3) northern Pacific coast; (4) the Rocky Mountain trench; and (5) northern Quebec and Labrador (Pybus 2001) (See Fig.2).

This liver fluke is an invasive species, which was introduced twice (or three times) to Europe with imported game animals (Kráľová-Hromadová et al. 2011). In the 19th century, it was introduced together with wapiti to the National Park La Mandria in Italy, near Turino (Balbo et al. 1987) from original habitats in North America, and the second time during the first half of the 1900s, it became apparent in Bohemia, part of the Czech Republic (Ulrich 1930), and this has persisted to the present day. Later, the next infection (probably the third introduction of the parasite as well) in Slovakia was reported in 3-year-old roe deer, near Gabčíkovo (Rajský et al. 1994). More recently, it has become established in Austria and Germany (Prosl 2001; Ursprung 2001; Vodňanský and Rajský 2001; Winkelmayer 2001; Winkelmayer and Prosl 2001; Rehbein et al. 2012), Hungary (Majoros and Sztojkov 1994), Croatia (Marinculić et al. 2002), and Serbia (Marinkovic and Nesic 2008). The liver fluke is currently spreading along the River Danube and can be expected to soon occur in new areas where there are no barriers to the movements of its definitive hosts (deer) (Špakulová et al. 2003a,b) (Fig.2B).

## Life Cycle

*F. magna* exhibits a life cycle that is similar to that of other trematodes, such as a closely related flukes, *Fasciola hepatica* and *Fasciola gigantica* (see Barker et al. 1993). Adults occur in pairs or groups within the fibrous pseudocysts in the liver of infected ruminants, and one pseudocyst may produce more than 4000 thick-walled operculate eggs per day (Swales 1935).

The eggs in moist external environments in temperatures between 15°C and 30°C mature into fully developed ciliated larvae – the miracidia. This phase of the life cycle lasts between 4 and 7 weeks. Eggs are only able to hatch in aerated water (Swales 1935; Campbell 1961). According to Pybus (2001), development depends on seasonal conditions of moisture, temperature, activity patterns of snails, and seasonal precipitation regimes or droughts. The miracidia produce proteolytic enzymes that weaken the operculum and thus allow hatching to occur (Pybus 2001). Development is inhibited in temperatures under 20°C or higher than 34°C (Campbell 1961). Erhardová-Kotrlá (1971) showed that eggs may successfully overwinter, but grow more slowly than those deposited during the spring and summer months. Swales (1935) showed that completion of development under summer field conditions takes approximately 35 days. The miracidia actively swim in water when searching for their intermediate hosts, water snails. If they do not find suitable hosts, they deplete their energy stores and perish (Erhardová-Kotrlá 1971). Employing an eye-spot organ that is sensitive to light, they display positive phototaxis, as well as being attracted by chemicals produced by lymnaeid snails, they are able to detect and infect intermediate hosts. In the aquatic snail, the fluke commences the asexual multiplication phase of the life cycle (Swales 1935; Erhardová 1961a,b; Erhardová-Kotrlá 1971). Miracidia penetrate tissues of the snail and search for the pulmonary sac, where they transform into sporocysts. The sporocysts are made up of germ cells, which subsequently produce the mother rediae. The rediae migrate primarily to the snail's hepatopancreas, although other organs may be infected as well. The daughter rediae are produced in the posterior regions of their bodies. The daughter rediae leave the mother rediae, and the process is repeated once again. The cercariae are subsequently produced within the second stage daughter rediae. Finally, the cercariae emerge from the bodies of the water snail hosts and then encyst on grass becoming infective metacercariae. The development within the lymnaeid snail lasts approximately 6–9 weeks (Swales 1935; Erhardová 1961a,b) and depends on such factors as temperature and host species. Pybus (2001) argues that a single miracidium, which has penetrated into a snail host, may eventually produce more than 1000 free-swimming cercariae.

The definitive hosts, ungulates, are infected when they ingest grass on which the cercariae have encysted. Erhardová-Kotrlá (1971) described two primary periods of transmission to the final host. The first is in the late summer and autumn, when grass bearing encysted metacercariae is grazed by herbivores, and the second period is in spring when the ungulates are foraging on fresh grass in wetland ecosystems. The metacercariae enter the gut of the definitive host, penetrate the intestinal wall, move in the abdomen cavity, and finally enter into the liver. They then migrate through the liver until they encounter another adult fluke. The immune reaction of the host is activated by the movements of the flukes in the liver, and this leads to the formation of as a pseudocyst in which the adult flukes are contained (Foreyt and Todd 1976; Foreyt et al. 1977). Within the pseudocyst, the flukes undergo further maturation and ultimately produce eggs. Erhardová-Kotrlá (1971) report that adult flukes may live as long as 5 years, and the prepatent period in ruminant hosts can be as short as 3 months or by as long as 7 months (Foreyt and Todd 1976).

## Spectrum of Intermediate Hosts

Intermediate hosts of *F. magna* are clearly as important for the parasite as the definitive host. Any break in the chain of transmission to these two host types would invariably prevent the fluke from completing its life cycle. The giant liver fluke now is found in both North America and Europe. In the North America, aquatic snails from the genus *Lymnaea* (subfamily Lymnaeinae; family Lymnaeidae) are reported as suitable intermediate hosts for *F. magna* (Foreyt 1981). In Europe, the same family Lymnaeidae and subfamily Lymnaeinae occur with genus name *Galba*. It has a worldwide distribution, and species in it exhibit great diversity in shell morphology, which is caused by ecophenotype plasticity. Family Lymnaeidae is important in terms of veterinary and human medicine, because species in it represent important intermediate hosts for members of the families Fascioloidae, Schistosomatidae, and Echinostomatidae, all of which include important human parasites (Correa et al. 2011). Species in the genus Galba are mostly confined to the peripheral zones of open drainage furrows and prefer habitats surrounding springs on hillsides, areas along the banks of rivers and ponds, and drainage ditches (Rondelaud et al. 2007). They are typically small aquatic snails, with shell heights in the range of approximately 5–11 mm and 3–7 mm in width. These snails are hermaphrodites and thus reproduce both by self-fertilization and outcrossing. Under benign environmental conditions, *G. truncatula* can grow up to 1 mm per week (Vignoles et al. 2003). Mekroud et al. (2002) studied the habitat of *G. truncatula* in northeastern Algeria and found that the highest snail densities were observed between December and March and the lowest between June and September, when the highest temperatures occur in this area. Snails from the autumn-born generation can overwinter and reproduce the following spring, ultimately dying in May or June just prior to the loss of wetland habitats due to dry conditions. On the other hand, the spring-born generation can estivate and lay eggs in autumn before dying at the end of the year (Mekroud et al. 2002).

Snails exhibit different susceptibility of infection with miracidia from different geographical areas (Barbosa 1961). Erhardová-Kotrlá (1971) found that miracidia can more easily penetrate into the snail in shallow warm waters than in cold, deeper waters. Well-fed snails are more likely to be infected than poorly nourished snails (Kendall 1949). North American snails are more resistant to infection by the liver fluke (Erhardová-Kotrlá 1971), perhaps because of a longer coevolutionary arms race between American snails and parasites. Aquatic snails can be infected with over than 600 cercariae as shown by Swales (1935) in *L. parva*. Infected snails exhibit retarded growth and castration, and heavily infected snails may die (Erhardová-Kotrlá 1971). Infection in snails can be verified by the presence of gymnocephalous cercariae released from captive or crushed snails (Pybus 2001).

### North America

*Lymnea truncatula* is a widespread species found over much of the world. For instance, it has been reported from North America, Europe, Africa, West, South and North Asia (Cywińska 2005). In North America, five snail species are naturally infected by *F. magna*, and these include *L. modicella, L. caperata, L. bulimoides, L. parva,* and *L. palustris nuttalliana* (Špakulová et al. 2003a,b). Family Lymnaeidae is distributed over North America from the Arctic Ocean to the Isthmus of Panama (Baker 1911; Dunkel et al. 1996 see Table1).

**Table 1:** Distribution of intermediate hosts of *Fascioloides magna* based on Baker's (1911) division of North American regions embracing natural drainage areas, and after Dunkel et al. (1996)

Intermediate hosts	Locality
*Lymnea bulimoides*	Californian (the coastal portion of Oregon and California), Texas
*Lymnea caperata*	Canadian (system of the St. Lawrence, All of Quebec and Ontario south of the Height of Land and Island of Anticosti); Hudsonian (western part of Ungava, southeastern part of Mackenzie, the whole of Keewatin, eastern Athabaska, the whole of Saskatchewan, Assiniboia, Manitoba, the southern part of Alberta, Ontario and Quebec northwest of Height of Land, the northern part of Minnesota and the eastern part of North Dakota); Yukonian (system of the Yukon river and north Alaska); Columbian (the southeastern part of British Columbia, the whole of Washington and Idaho, the western part of Montana and the eastern part of Oregon); Coloradoan (the southern part of Nevada and Utah, the western parts of Colorado and New Mexico, the southeastern part of Wyoming, the southeastern California, the northwestern part of Mexico and the whole of Arizona); Great Basin (the desert and arid regions of Nevada, Utah, Oregon and California); Californian (the coastal portion of Oregon and California); Upper Mississippian (south of the Height of Land in southern Canada and the Great Lakes, all of the territory between the Rocky Mountains on the west and the Appalachian Mountains on the east); Nova Scotian (the southeastern part of New York, the east-central portion of Pennsylvania, northern New Jersey, Virginia, the greater part of Maryland, the whole of Nova Scotia, New Brunswick, Newfoundland)
*Lymnea parva*	Canadian; Hudsonian; Columbian; Coloradoan; Rio Grandian (southern part of Colorado, nearly the whole New Mexico, the western part of Texas, northern and central part of Mexico); Upper Mississippian; Lower Mississippian (south of the Arkansas and Tenessee river); Nova Scotian
*Lymnea modicella*	Las Vegas valley, Idaho, Washington, Montana
*Lymnea palustris nuttalliana*	Searles Lake, Washington, Alberta

### Europe

*F. magna* has three persistent foci in Europe: in the natural park La Mandria in Italy, in southern and central Bohemia, and in the territory of the Danube floodplain forest (Špakulová et al. 2003a,b). In all of these areas, only two naturally infected snails species have been confirmed, *Galba truncatula* and *Radix* (syn. *Lymnaea) peregra* (Erhardová-Kotrlá 1971; Faltýnková et al. 2006). Although *Radix* (syn. *Lymnaea) peregra* is more widespread within Europe, under natural conditions, Faltýnková et al. (2006) found only six infected snails of 7 277 collected in total. *G. truncatula* is mainly found on periodically flooded, sandy–muddy habitats as well as in running waters (Reckendorfer and Schaefer 2003) and is a widespread species (Madhyastha 2012). This snail is also important in terms of being an intermediate host of *Fasciola hepatica*, a serious parasite of animals and humans (Correa et al. 2011). According to Slusarski (1955), there are other possible intermediate hosts of the giant liver fluke, for example *L. stagnalis*, but this has not been demonstrated in natural conditions. Moreover, other snail species such as *R. ovata* and *S. palustris*, may be invaded by *F. magna*, but endogenous development was arrested at the mother redia stage and the snails subsequently died (Erhardová-Kotrlá 1971). The snails *Succinea oblonga, S. putris, L. stagnalis, Stagnicola palustris,* and *Physa acuta* were not successfully infected under laboratory conditions (Erhardová-Kotrlá 1971).

The spectrum of intermediate hosts in North America is larger, because *F. magna* is native to North America and has therefore had time to adapt to the snail fauna there. Therefore, it can be assumed that also in Europe, the giant liver fluke over time may adapt to other snails in the family Lymnaeidae (Rondelaud et al. 2007).

## Spectrum of Definitive Hosts

The primary definitive host species of the giant liver fluke in the native range is the white-tailed deer (*Odocoileus virginianus*) (Pybus 2001). However, the spectrum of hosts is much broader. These hosts are divided into three groups: (1) definitive hosts, (2) aberrant hosts, and (3) dead-end hosts (Pybus 2001). In North America, the definitive host group includes white-tailed deer (*O. virginianus*), wapiti (*Cervus canadensis*), caribou (*Rangifer tarandus*), and black-tailed deer (*O. hemionus hemionus*), whereas in Europe, it includes red deer (*C. elaphus*) and fallow deer (*Dama dama*) and roe deer (*Capreolus capreolus*), all are tolerant to infection and where the endogenous phase of the development cycle runs through a typical course (Erhardová-Kotrlá 1971; Pybus 2001).

The infection in definitive hosts entails the migration of immature flukes, during which time they search for other flukes and congregate in thin-walled fibrous pseudocapsules, which typically contains two or more adult flukes (Foreyt et al. 1977). Migration of immature flukes does have serious consequences on the tissue of the host liver and the creation of the pseudocapsule within the hepatic parenchyma (Foreyt and Todd 1976). Cysts are filled with dark-green liquid containing degraded bilirubin and fluke (Swales 1935). The physiological effects of high egg production may be highly deleterious to the host, because the eggs may block bile ducts or stimulate the production of a pseudocyst, which may rupture the hepatic parenchyma via pressure atrophy (Pybus 2001). This has been reported in South Carolina, where hepatic destruction was found to be associated with 125 individuals of *F. magna* recovered from the liver of a white-tailed deer (Purgslove et al. 1977). Similar damage was described from the infection of 500+ flukes in the liver of a wapiti in Alberta (Butterworth and Pybus 1993). Death is most often associated with large volumes of blood loss, ruptured livers, or an inflammation of the peritoneum. In Europe, roe deer and fallow deer show the highest mortality as a result of fascioloidosis (Záhoř 1965; Rajský et al. 2002), and different signs of infection with *F. magna* in cervids include reduced weight gain, reduced quality of antlers, or a bad rut course (Erhardová-Kotrlá 1971).

Infection in aberrant hosts such as sheep, goats, and bighorn sheep has fatal consequences which contrasts with effects on suitable definitive hosts (Pybus 2001). In suitable definitive hosts, immature flukes must migrate in order to find other flukes prior to being enclosed in the pseudocyst. However, in aberrant hosts, excessive wandering and a lack of encapsulation result in severe damage to the liver and eventual death of the host (Foreyt and Todd 1976). Furthermore, immature flukes penetrate into various abdominal and pleural organs, leading to hemorrhagic cavitation, interlobular edema, or fibrotic septae (Stromberg et al. 1985). Adult flukes in aberrant hosts occur only very rarely as well as their eggs (Erhardová-Kotrlá 1971).

Large ungulates such as moose (*Alces alces*), bison (*Bison bison*), sika deer (*Cervus nippon*), llama (*Lama glama*), horse, pig, and cattle are classified as dead-end hosts (Foreyt and Todd 1976). Unlike aberrant hosts, the immune system of dead-end hosts also creates a pseudocyst in the hepatic parenchyma, but these accumulate immature eggs because they do not have an outlet into the environment (Záhoř 1965). Foreyt and Todd (1974), however, suggested that eggs may be found in the feces of cattle with severe infections or because of a rupture of the pseudocyst.

Here, we describe three of the most important definitive host species within North America and two species in Europe, with emphasis on their historical and present ranges.

### North America

#### *Odocoileus virginianus* Zimmermann, 1780

The white-tailed deer, also known as Virginia deer, is native to North America and is found in open woodland and field habitats from southern Canada through Mexico into South America. This species has also been introduced into New Zealand and European countries including Italy, Finland, Czech Republic, and Serbia (Gallina and Lopez Arevalo 2008). In North America, it inhabits five biotic regions, with 16 subspecies of the white-tailed deer thus far being described (Wilson and Reeder 2005). However, thus far 38 subspecies of whitetail in North and South America have been described, although DNA testing is showing that many of the deer now listed as subspecies are actually just locally adapted versions of the originally described subspecies (Wilson and Reeder 2005).

*O. virginianus* belongs to the family Cervidae, which also include the Old World deer. According to Stehli and Webb (1985), they probably entered North America from Asia through the Bering Straits during the early Pliocene. The species continued to expand to the south and east, eventually finding its way into South America via the Panama land bridge connecting this continent with North America around 2.5 million years ago (Stehli and Webb 1985). This hypothesis is confirmed through the fossil record for this genus from the early Pliocene (Kurten and Anderson 1980).

**Figure 3:**
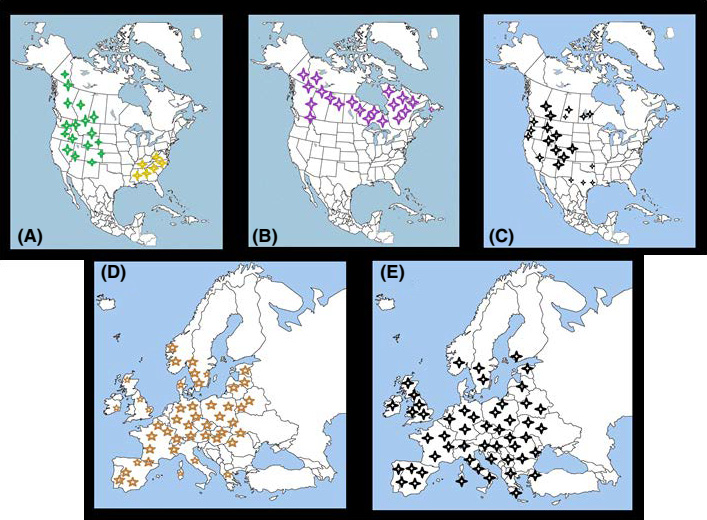
(A) Range map of white-tailed deer (*Odocoileus virginianus*) (yellow stars; a small population on the right side) and mule deer (*Odocoileus hemionus*) (green stars; a large population on the left side). (B) Range map of caribou (*Rangifer tarandus*). (C) Range map of wapiti (*Cervus elaphus canadiensis*). (D) Range map of red deer (*Cervus elaphus*). (E) Range map of fallow deer (*Dama dama*).

According to Pybus (2001), the population of this deer declined steadily after 1500 which coincides with the arrival of European settlers. The United States National Park Service estimates that between 23 and 40 million white-tailed deer inhabited North America before the arrival of Europeans. In the late 1800s, market hunting reduced the whitetail population to an all-time low of approximately 500,000 animals, and they disappeared completely in some areas of the eastern United States. In 1908, 41 states passed laws protecting the species, enabling numbers to rebound, and as a consequence by 1970, whitetail populations were growing steadily across the lower 48 states as well as in Canada. The elimination of apex predators, including the timber wolf and mountain lion, from vast portions of their original ranges also enabled deer populations to increase rapidly. Today, an estimated 14 to 20 million are believed to inhabit the United States alone, and in many areas of the eastern U.S., populations have soared to previously unattained levels (Swihart and DeNicola 1997) (See Fig.3A, but only for two confirmed subspecies of *F. magna*). As a result, the deer is now considered to be a pest in some areas due to heavy over browsing of native vegetation.

#### *Rangifer tarandus caribou* (Linnaeus, 1758)

The present North American caribou taxonomic classification is built on mtDNA and recognizes four subspecies in North America: *Rangifer tarandus caribou, R. t. granti, R. t. groenlandicus* and *R. t. pearyi* and two subspecies in Europe and Asia: *R. t. platyrhynchus, R. t. fennincus* (Eger et al. 2009). Nevertheless, some authors acknowledge the presence of a fifth subspecies of caribou in North America, on the Queen Charlotte Islands, *R. t. dawsoni*, but based on mtDNA and microsatellites, this subspecies is not necessarily distinct from others (Byun et al. 2002). On the other side, based on ecological characterization according to Flagstad and Røed (2003), North American caribou can be classified into three major groups: the woodland form, the continental tundra form, and the high Arctic Island form, each showing distinct morphological characteristics.

Flagstad and Røed (2003) inferred refugial origins of *R. tarandus* from mitochondrial DNA sequences and divided the species into three haplogroups. The first haplogroup is of pure Eurasian origin, which includes individuals from Fennonscandia and Russia. The second haplogroup is distinct from the others, and all haplotypes belonging to this group are found among the more southerly distributed woodland caribou in parts of southern and central Canada, where they inhabit boreal forest ecosystems. The third haplogroup involves North American and Eurasian subspecies (*R. t. grantii, R. t. groenlandicus, R. t. pearyi,* from North America, and *R. t. tarandus, R. t. platyrhynchus, R. t. fennincus* from Eurasia). And as reported by Flagstad and Røed (2003), the third haplogroup could include individuals from an ancestral glacial population that dispersed across vast areas of tundra in Eurasia and eventually came into North America across the Bering land bridge some 115,000 years ago (but see Courtois et al. 2003).

The evolutionary history and biogeography of North America caribou were constructed by Eger et al. (2009) using mtDNA. They sequenced 184 *Rangifer* from 16 localities in Alaska, the Canadian arctic (central arctic, including Nunavut, Nunavik, Nunatsiavut, and N W Territories), southern Yukon, Ontario, Quebec, and from Newfoundland. Phylogenetic analyses of the data showed that caribou from Ontario and Quebec are related to caribou from Newfoundland, but the Newfoundland population was separated from the Ontario and Quebec populations approximately a century ago (Eger et al. 2009). Furthermore, this group is probably of a very old lineage, separated from most other caribou by at least 80,000 years. Populations from Alaska and the Yukon are included as one, and those from the central Arctic appear to be derived from a Banks Island refugium. However, there is strong evidence for a second old lineage in the high Arctic from Bathurst Island, which is genetically distinct from the others and this line probably diverged at a much earlier date (Eger et al. 2009).

The Quebec and Labrador populations declined considerably in the late 19th century, and this process still continues (Courtois et al. 2003). Moreover, many forest-dwelling populations, which persisted during the 1950s and 1960s, have now also disappeared (Courtois et al. 2003). Caribou herds have occurred in northwestern Ontario until at least very recently (Seip 2010). Kelsall (1984) considered Ontario's woodland caribou “vulnerable.” The size of the western Canadian population has been based on various estimates of the population size or status of caribou in Ontario. Populations in Alberta have rapidly declined over many years owing to many of the same factors that hinder populations elsewhere on the continent (Bradshaw et al. 1997). It is of profound concern that numbers of woodland caribou in Alberta are estimated to have declined from approximately 9000 in the mid-1960s to <2000 at present (Edmonds 1988). The woodland caribou in British Columbia is divided into three groups based on distribution: the mountain, northern and boreal ecotypes (Heard and Vagt 1998), and the populations are estimated to contain an estimated 16,500 individuals (Spalding and Branch 2000). See actual range map (Fig.3B).

#### *Cervus elaphus canadensis* (Linnaeus, 1875)

*Cervus elaphus canadensis*, also known as wapiti or elk, represents another suitable obligate host for *F. magna* (Kennedy et al. 1999). In North America, the elk once ranged across southern Canada and over most of the United States, but loss of habitat and excessive hunting pressure eliminated the species over much of its range, particularly in the east (Fig.3C). Pitra et al. (2004) derived the term “wapiti” as the subspecies of *C. elaphus* in eastern Asia, Siberia, and North America, while “elk” is more common in North America. Therefore, Polziehn and Strobeck (2002) used mtDNA to determine the phylogeny of wapiti with respect to European red deer, and they separated them into two clades which represented two independent species – the European *C. elaphus* and the North American *C. canadensis*. Until recently, six subspecies of *C. canadensis* have been described and which inhabited North America in historical times. But only four of them are still extant, including the Roosevelt (*C. c. roosevelti*), Manitoban (*C. c. manitobensis*), Tule (*C. c. nannodes*), and Rocky Mountain (*C. c. nelsoni*), and two extinct subspecies the Eastern elk (*C. c. canadesis*) and Merriam's Elk (*C. c. merriami*) (Ludt et al. 2004).

Fossil records place Cervinae in the early Pliocene (Lydekker 1898; Comincini et al. 1996). Kuwayama and Ozawa (2000) suggest that the times at which European red deer, wapiti, and sika deer diverged occurred around the Plio-Pleistocene boundary 1.60 Mya. In the same study, they estimated the divergence time between the Asian wapiti (*C. e. kansuensis, C. e. xanthopygus*) and the North American wapiti (*C. e. canadensis*) by means of mitochondrian DNA sequences as 0.28 Ma, which was confirmed by the first fossil record of wapiti in Alaska from the Illinoian glacial period (0.26–0.15 Ma) (Geist 1971; but see Guthrie 1966).

Elk is one of the most popular game animals in North America, and therefore, it has a rich history in terms of hunting and reintroductions into habitats from which it was extirpated. *C. elaphus canadensis* has recently been reintroduced into the states of Kentucky, North Carolina, and Tennessee, and from these areas, it has migrated to the neighboring states Virginia and West Virginia where it is doing well in Appalachian deciduous forests. The species is commonly now seen at Great Smokey Mountains National Park in Tennessee. Reintroductions have also been made in Pennsylvania, Michigan, Wisconsin, and Alaska. Between 1981 and 1985, Arkansas, Colorado, and Nebraska also released elk into suitable habitats. In Canada, wapiti reached their maximum geographical range before 1800, but the impact of hunting was larger than populations could sustain and the wapiti soon disappeared from eastern provinces (Ontario and Quebec). Currently, small populations survive in Canada in the provinces of Saskatchewan, Manitoba, British Columbia as well as on Vancouver Island and the Yukon (Fanti and Catuneanu 2010; Smits 1991).

### Europe

#### *Cervus elaphus* Linnaeus, 1758

The remarkable morphological diversity within the European genus *Cervus* (Red deer) has given rise to about 15 species and numerous subspecies (Gyllensten et al. 1983). Several red deer subspecies native to northern Europe have previously been recognized, for example, the Swedish nominate *Cervus elaphus elaphus*, the Norwegian *C. e. atlanticus*, the British *C. e. scoticus*, and the continental *C. e. hippelaphus*, and *C. e. germanicus*. Nevertheless, morphological and genetic analysis (Lowe and Gardiner 1974; Gyllensten et al. 1983; Ludt et al. 2004) provided evidence only for the existence of four subspecies in Europe (*C. el. barbarus*, *C. el. hippelaphus, C. el. elaphus*, and *C. el. corsicanus*). However, the question of subspecies diversification is still under investigation.

The fossil record shows that Red deer inhabited both interglacial/woodland and glacial/steppe faunal complexes of the Middle Pleistocene (Lister 1984; Kahlke 1992; von Koenigswald and Heinrich 1999; von Koenigswald 2002; Benecke 2004). This suggests that the species had a broad tolerance of different habitats (Sommer et al. 2008), which is also supported by the evidence of fossils found in Great Britain (Lister 1984), Spain (Altuna 1972), France (David 1985), Moldavia (David et al. 2003), and Greece (Olsen 2000).

The origin of the genus *Cervus* may be lying in Central Asia (Ludt et al. 2004). Moreover, molecular phylogeny suggests three independent preglacial immigration routes from Asia to Europe (Ludt et al. 2004; Skog et al. 2009). Moreover, red deer from Sardinia/Corsica, North Africa, and southern Spain diverged before the western and eastern European populations evolved from a common ancestor, and the eastern and western European populations descended from glacial refugial populations in the Iberian Peninsula and the Balkans, respectively (Skog et al. 2009).

Unlike the other species (see above), *C. elaphus* has increased in abundance throughout Europe over the last century (Gill 1990; Gordon 2009) which was consequence that in some countries, management strategies have shifted from species protection to population control as a result of the increasing numbers of this deer (Brown et al. 2000; Côté et al. 2004) (Fig.3D).

#### *Dama dama* Linnaeus, 1758

At present, there is only one species of Fallow Deer (*Dama dama*) which is broken down into two subspecies: the European Fallow (*Dama dama dama*) and the Persian Fallow (*Dama dama mesopotamica*) (Wilson and Reeder 2005). Alternatively, other authors consider *D. d. mesopotamica* to be a different species, based on a major study on the evolution and phylogeny of old world deer (Pitra et al. 2004; Masseti et al. 2008).

*Dama dama* is a Western Palearctic species, whose original range is still unclear (Masseti et al. 2008). Current knowledge suggests that Turkey and southern Europe (southern Italy, Sicily, and Balkan) were the postglacial refuges of the species (Masseti et al. 2008). Moreover, fossils of fallow deer on the island of Rhodes go back to Neolithic times (Mertzanidou and Legakis 2004).

Later, the species was introduced to the western Mediterranean by the Phoenicians and to central and northern Europe by the Romans and Normans. However, most of the currently existing populations in Europe result from much more recent introductions (Masseti et al. 2008). Nevertheless, the distribution in Europe is still much more scattered and patchy because most of the populations are constrained in their movements by fences (Chapman and Chapman 1980).

Although it has become extinct over much of its former range, most introduced populations in Europe are presently considered to be stable (Apollonio 1989) (See Fig.3E). Consequently, the IUCN lists the species under the category of “least concern.”

## Domestic Ruminants and Experimental Infections

Sheep, goats, bighorn sheep, rabbits, and guinea pigs are not definitive hosts for *Fascioloides magna*. Instead, they are aberrant hosts, in which the fluke has lethal effects leading to host death (Pybus 2001). This is due to the unrestricted migration of liver flukes in the hepatic parenchyma and other organs such as the lungs and abdominal cavity. Aberrant hosts die usually within 5 months of initial infection, and death is associated with acute peritonitis, extensive hemorrhage, and a diffuse fibrosis throughout the liver (Foreyt 1992). Goats and sheep apparently do not exhibit previous clinical signs of infection (Foreyt 1996), but in some cases, infected animals show signs of lethargy shortly before death (Foreyt 1990). Mature flukes appear only rarely, but few eggs are typically released prior to the death of the host (Erhardová-Kotrlá 1971).

Under laboratory conditions, metacercariae of flukes were inoculated into guinea pigs and domestic swine. The guinea pigs died within 2–4 months of inoculation, whereas trematodes were found in the hepatic parenchyma and peritoneal cavity of infected animals. Age resistance to infection has been shown in domestic swine, where animals ∽10 weeks of age were more resistant to flukes than 2-week-old animals (Foreyt 1979). Conboy and Stromberg (1991) studied experimental infections in guinea pigs and found that these animals are suitable as models for *F. magna* infection in sheep, because the pathologic process was observed in the lungs and pulmonary arteries along with the pattern of hepatic and pulmonary lesions, possibly leading to use of the cardiovascular system as a pathway for dissemination. Experimental inoculation of 250 metacercariae into seven goats resulted in the death of six several months after inoculation, and the infection exhibited similar effects as demonstrated in cattle, white-tailed deer, and sheep (Foreyt and Leathers 1980).

Natural infection of sheep when sharing pasture habitat with Columbian white-tailed deer was described in Oregon by Foreyt and Hunter (1980) as well as in Texas cattle, where *Fasciola hepatica* and *Fascioloides magna* were for this first time found together in the one animal (Foreyt and Todd 1972). Infections in domestic dead-end hosts such as bovids, suids, llamas, and horses are characterized by excessive fibrosis, thick-walled encapsulation of the parasite within the hepatic parenchyma, and black pigmentation in the abdominal cavity with only a rare occurrence of the eggs (Pybus 2001). An increasing rate of fascioliasis was detected in cattle from Montana and the south central region corresponding to the Yellowstone River drainage system (USDA). In 1990, there was a 12% increase in liver infection compared with 1973 (Knapp et al. 1992). Migaki et al. (1971) identified the first natural incident of giant liver flukes in pigs, and Schwartz et al. (1993) reported the first event of *F. magna* in a feral pig shot by hunters in Texas. In Minnesota, the first natural infection of a 25-year-old mare was reported (McClanahan et al. 2005).

One natural infection of llama was also been reported in Minnesota, where five immature flukes were recovered from an individual which exhibited a response similar to that exhibited by cattle (Conboy et al. 1988). Foreyt and Parish (1990) confirmed this in an experimentally infected llama, where the pathological effects of infection were similar to that seen in cattle and moose, but not as severe as in sheep and goats. Infection exhibited no clinical signs. Mature flukes appeared in the liver, but eggs were not produced in the feces (Foreyt and Parish 1990).

Transmission to domestic ruminants takes place in enzootic areas where pastures are shared with wild ungulates (Foreyt and Hunter 1980). Transfer of the fluke to domestic sheep and cattle herds can lead to large economic costs due to mortality of the animals and destruction of infected livers. Despite the native status of the giant liver fluke in areas of North America, there does not, however, appear to be a significant problem in the livestock industry there (Pybus 2001). Unlike processes characterizing the infection cycle in North America, until the present, there has been no evidence for ruminant's infection in Europe, probably because European wild ungulates do not share pasture habitats with domestic ruminants.

## Discussion

The current study provides a comprehensive overview of the biology and phylogeography of the giant liver fluke, *F. magna*, and both its intermediate (snail) and definitive (wild ungulate) hosts in both its native North American and invasive European ranges. For *F. magna* to survive and persist in habitats on both continents, it is vitally important that it is sympatric in space and time with both its intermediate and definitive hosts (Erhardová-Kotrlá 1971). Despite being native to North America, there are major constraints in this regard. Currently, there are only five known major centers of distribution (or “foci”) of *F. magna* in North America (Fig.2A) (Pybus 2001). However, suitable intermediate snail hosts (Lymnaeidae) are common and ubiquitous in wetland habitats over most of the continent (See Table.1) (Cywińska 2005), and suitable definitive hosts are also found over most of North America. It is therefore difficult to understand why the fluke has not spread over a much larger part of the continent given that appropriate biotic conditions appear to be widespread. It may be that certain areas of realized distribution contain more optimal (e.g., climatic, habitat related etc.) abiotic conditions than areas of potential (=unrealized) distribution. Moreover, each of the populations may have become locally adapted to hosts and ecosystems where they are found, and thus, we are seeing speciation in its early stages. For example, flukes found in northern Quebec may be adapted to colder conditions in boreal habitats and to caribou which are the only suitable definitive hosts found there. Alternatively, in the southern United States, flukes enjoy a much more benign climate and probably specialize on white-tailed deer as definitive hosts. Genetic screening of flukes from different populations could also help to elucidate differences in their biology as a result of adaptation to local conditions.

By contrast, in Europe, there are only three foci which are known to have established in a temporal sequence (Fig.2B) (Kráľová-Hromadová et al. 2011). The first introduction occurred with wapiti in Italy over a century ago and persists to this day (Marinculić et al. 2002). However, the fluke has been unable to expand its range from these foci, because the wapiti have been constrained in their movements by fences which have acted as physical barriers to dispersal (Kráľová-Hromadová et al. 2011). The habitats in which the wapiti are constrained also include suitable wetland habitats for *G. trunculata* and *Radix* (syn. *Lymnaea) peregra* (in this case, only 6 of 7 277 samples were found infected)*,* which thus far are the only known Palaearctic suitable intermediate host for *F. magna* in nature (Faltýnková et al. 2006; Vignoles et al. 2003, 2006). Consequently, even within a relatively small geographical area, the fluke is able to survive because both biotic and abiotic conditions facilitate it. Although the host range in Europe is more limited than in North America, there is considerably more overlap among the distributions of them than among the North American counterparts. This is because the second and third introductions have occurred in the proximity of the Danube River (the Czech-Danube foci), along which the fluke is spreading due to suitable wetland habitats along the river for both host types and because of human-assisted transport (e.g., ships and other water transport) (Špakulová et al. 2003a,b). There is high possibility that the fluke will continue to expand its range if it enters other riverine systems in Europe, which is confirmed by its recent occurrence in Serbia (Marinkovic and Nesic 2008).

Another important factor in this regard is that the three European foci also exhibit very broad geographical overlap with roe deer (data not shown), fallow deer, and red deer (Fig3D, E). Indeed, all three deer species have very broad ranges and occur in multiple habitats in Eurasia (Hewison et al. 2001; Bruinderink et al. 2003; Sommer et al. 2008). In contrast, the fluke does not appear to be spreading in North America, in spite of being native there and the fact that suitable deer and snail species are widespread and abundant over many habitats (Pybus 2001). The difference may be attributable to the fact that the fluke is engaged in a strong coevolutionary arms race with North American hosts, in which resistance has evolved in many intermediate and/or definitive hosts there over many millennia, and although there is high prevalence of parasite in cattle, this host is not suitable for accomplishment of the life cycle (Pybus 2001). In Europe, native snails and deer have no evolutionary history with the fluke which has been able to infect them with less physiological resistance (Kráľová-Hromadová et al. 2011). This is an area that is ripe for future investigations.

**Figure 4:**
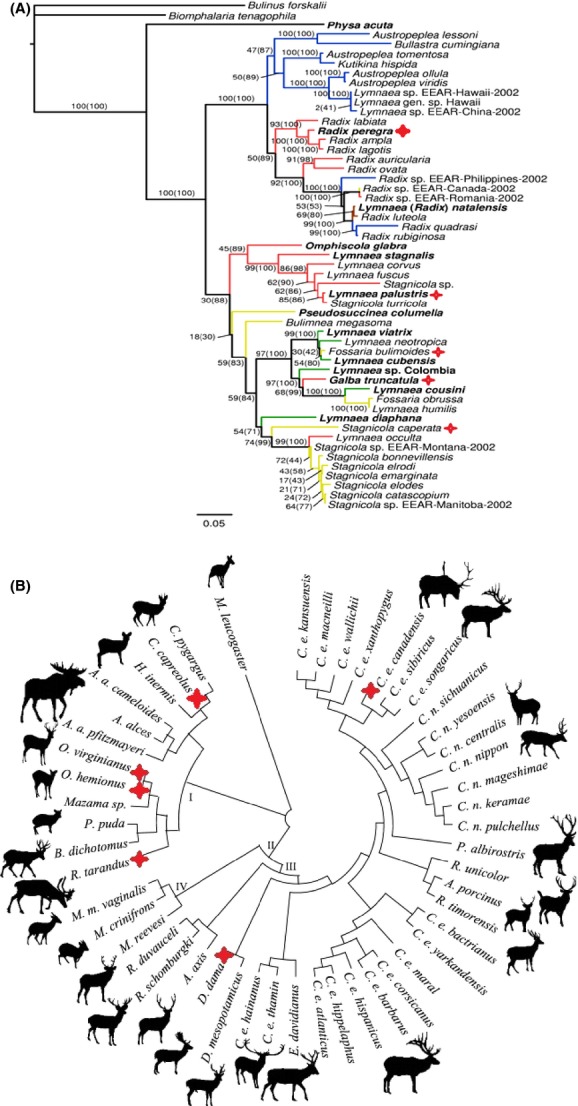
(A) Phylogeny of family Lymnaeidae by Correa et al. (2011) highlighting known intermediate hosts confirmed under natural conditions. Suitable intermediate host are *Radix* (syn. *Lymnaea*) *peregra, Lymnaea palustris, Fossaria bulimoides, Galba truncatula* and *Stagnicola caperata*. (B) Phylogeny of family Cervidae by Pitra et al. (2004) highlighting suitable definitive hosts confirmed under natural conditions (currently no study has described subspecies of *Cervus elaphus* as a definitive host in Europe, thus this is not highlighted). Proper definitive hosts *Fascioloides magna* are *C. capreolus, C. e. canadensis, Dama dama, Rangifer tarandus, Odocoileus hemionus,* and *O. virginianus*.

The phylogeny (and hence physiological equivalence) of the various native and exotic intermediate and definitive hosts is critically important in determining their suitability as hosts for *F. magna* (Parker et al. 2001). The five North American *Lymnaea* intermediate hosts species are closely related to the European snail host, *G. trunculata* (See Fig.4A), which was proved by successful spreading of *F. magna* via Danube river (Marinkovic and Nesic 2008). Not only are the snails closely related, but they inhabit similar wetland habitats in both continents where suitable definitive hosts also forage for succulent vegetation (Trouve et al. 2005). Irrespective of species, the infected snails are castrated and are thus not able to reproduce, meaning that the different generations of the fluke need to find new healthy snail hosts to persist in the environment (Chapuis 2009). This is not the case with suitable definitive hosts, which can be reinfected by different generations of *F. magna* (Pybus 2001).

The deer family (Cervidae) is one of the largest (about 40 species worldwide), most abundant, and widespread families of ruminant ungulates (Ruminantia) (Kuznetsova et al. 2005). The various species and subspecies of native North American exhibit two levels of phylogenetic overlap with the three species of European deer which now act as definitive hosts for giant live fluke in the new range (See Fig.4B). This is confirmed by the close phylogenetic relationship that exists between the Cervidae and Bovidae (Ludt et al. 2004). Moreover, based on Vrba and Schaller (2000), a conservative fossil record calibration placed the most common ancestor for Cervidae and Bovidae 25 million years ago (but see Ludt et al. 2004), which may play an important role in defining a definitive host for *F. magna*. North American wapiti (*Cervus el. canadensis*) is the same species (but different subspecies) of European red deer (*Cervus elaphus*). The fallow deer, *D. dama*, is phylogenetically close with species in the genus *Cervus* (Pitra et al. 2004), facilitating an easy switch between the two definitive hosts. *Odocoileus* species and *R. tarandus* exhibit a strong phylogenetic affinity (Kuznetsova et al. 2005). This facilitates a clear physiological link for the liver fluke.

As discussed by Harvey et al. (2012), physiological equivalence among hosts of parasites is vitally important in determining the suitability (or unsuitability) of hosts for their parasites. This may be due to immunological parameters (Strand and Pech 1995) or other biological aspects of a host's internal milieu that are phylogenetically conserved. Therefore, the physiological equivalence is an important prerequisite for the success of novel resource–consumer interactions and ecological fitting (Harvey et al. 2012). Moreover, the ability of species to exploit novel habitats is often correlated with the dimensions of its realized niche (contrast to fundamental) in nature, and subsequently, ecological and evolutionary specialization is usually associated with a reduction in niche breadth, thus limiting the possibility of a species or species association with colonize novel habitats or ecosystems. On the other hand, many species possess traits that enable them to colonize and proliferate in many different habitats (Parker et al. 2001). As the invasion ecology literature shows, many of the most successful exotic animals and plants possess highly generalized suites of traits under a broad macro-evolutionary umbrella that broaden the number of ecotypes in which they can colonize and spread. Thus far, most successful and ecologically and economically invasive plant and animal species have been shown to exhibit “broad-spectrum fitting” traits (e.g., “r-selection”) which enhances their ability to establish and spread in novel habitats (Lockwood et al. 2013).

This study, on the other hand, provides a more refined example of a parasite species with highly refined ecophysiological requirements that nevertheless has overcome numerous ecological and physiological challenges to establish as a potentially major parasite of three native species of European ungulates. Most importantly, it shows that species exhibiting even quite complex life histories can become successful invaders in novel habitats, an example of “multiple-level ecological fitting.” (Janzen 1985; Brooks et al. 2006; Malcicka M, Agosta S, Harvey JA in prep.).
